# Validity, sensitivity and specificity of a measure of medication adherence instrument among patients taking oral anticoagulants

**DOI:** 10.1002/prp2.1113

**Published:** 2023-10-27

**Authors:** Mariana Dolce Marques, Rafaela Batista dos Santos Pedrosa, Henrique Ceretta Oliveira, Maria Cecília Bueno Jayme Gallani, Roberta Cunha Matheus Rodrigues

**Affiliations:** ^1^ School of Nursing University of Campinas São Paulo Brazil; ^2^ Sciences infirmières Université Laval Quebec City Quebec Canada

**Keywords:** anticoagulants, medication adherence, nursing, self‐care, validity

## Abstract

Although self‐report instruments are currently considered a valuable tool for measuring adherence, due to their low cost and ease of implementation, there are still important factors that impact measurement accuracy, such as social desirability and memory bias. Thus, the Global Assessment of Medication Adherence Instrument (GEMA) was developed to provide an accurate measure of this construct. The aim of this study was to evaluate the properties of the measurement of the Global Evaluation of Medication Adherence Instrument (GEMA) among patients with chronic diseases. A methodological study was conducted in the public hospital of the state of São Paulo, Brazil. The adherence to anticoagulants as well as the international normalized ratio (INR) was assessed on 127 patients. Besides GEMA, two other instruments were used to assess adherence: the Morisky Medication Adherence Scale‐8 (MMAS‐8) and the Measurement of Adhesion to Treatments (MAT). The GEMA presented a satisfactory level of specificity (0.76) to identify adherents among those with a stable INR, low sensitivity (0.43) for the identification of non‐adherents among those with an unstable INR, and a Positive Predictive Value of 0.70. Positive and weak to moderate correlations were observed between the proportion of doses assessed with GEMA and the scores on the MMAS‐8 (*r* = .26 and *r* = .22, respectively) and the MAT (*r* = .22 and *r* = .30, respectively). The GEMA presented good practicality, acceptability, and evidence of specificity regarding the stability of the INR. The validity of the construct was partially supported by the relationship with self‐reported measures of adherence.

**Abbreviations**
COSMIN: Consensus‐based Standards for the Selection of Health Measurement Instruments
GEMA: Global Assessment of Medication Adherence Instrument
INR: International Normalized Ratio
MAT: Measurement of Adhesion to Treatments
MMAS‐4: Morisky Medication Adherence Scale‐4
MMAS‐8: Morisky Medication Adherence Scale‐8
NCDs: No Communicable Diseases
NPV: Negative Predictive Value
OAC: Oral Anticoagulants
PPV: Positive Predictive Value
TTR: Time in therapeutic range

## 1 INTRODUCTION

In the treatment of chronic no communicable diseases (NCDs), adherence to medication use has been associated with optimization of clinical outcomes, especially better disease control, reduction of hospitalizations, mortality, and health care costs.^1^, ^2^, ^3^ However, the percentage of medication nonadherence remains high.^4^, ^5^, ^6^

Medication adherence is one of the most complex self‐care behaviors in the treatment of NCDs.^7^ According to the middle‐range *Theory of Self‐care of Chronic Illness*, self‐care can be defined as a process of health maintenance by means of health practices and disease management, which can be applied to health and disease situations.^8^ In this context, the maintenance of self‐care refers to behaviors to maintain well‐being, health and physical and mental stability, such as smoking cessation, healthy food consumption, stress management, and medication adherence.

Nurses have a central role in promoting self‐care,^9^ especially regarding medication adherence, which implies an assessment and a decision on the need to intervene.^10^ However, the measure of this behavior has shown to be extremely complex.

There are several difficulties in measuring adherence.^1^ Although there is no consensus on a “gold standard”, different methods have been used to measure medication adherence and are classified as direct (direct observation of medication intake and biological measurements—serum drug concentration levels) and indirect (self‐report measurements, monitoring through electronic pill dispensers, pill counting, and pharmacy refill).^11^ Among the indirect methods, the most used measures are the self‐report instruments for measuring medication adherence given their ease of implementation, low cost, flexibility (time and mode of administration), and low burden on the respondent. However, self‐report instruments present potential disadvantages, especially social desirability and memory bias, which compromise the accuracy of this type of measure.^12^, ^13^

Several self‐report scales that measure adherence in chronic diseases are available in the literature.^14^, ^15^, ^16^ The *Morisky Medication Adherence Scale* (MMAS) consisting of four items (MMAS‐4)^17^ or of eight items (MMAS‐8)^18^ are the ones of the most frequently used tools.^15^, ^16^ However, reports of overestimation of adherence with the use of self‐reported measures have been frequent.

In order to provide an accurate measure of medication adherence, the *Global Evaluation of Medication Adherence Instrument* (GEMA)^19^ was developed based on the previous study.^20^ The GEMA assumes that adherence is a complex and dynamic behavior, defined as “taking medication for treatment, exactly as prescribed, which means, taking it every day, at the time and amount indicated, remembering the care needed when taking it”,before and after meals and/or at bedtime”.^19^ This instrument proposes, in addition to the measurement of the proportion of doses, the assessment of self‐care associated with medication intake. In addition, to reduce memory bias, GEMA proposes to access the memory to retrieve the proportion of doses taken in different periods, up to the period of interest for measurement: that is, the month prior to the interview.

This new instrument seeks to fill the gaps in the literature regarding the imperfections of self‐report measures.^1^, ^13^ Although the GEMA has been used in previous studies,^19^, ^21^, ^22^, ^23^, ^24^, ^25^ to our knowledge, no study investigated its properties of the measurements. The objective of this study was to evaluate the measurement properties of the GEMA when administered to patients taking oral anticoagulants (OAC) in an outpatient follow‐up. The feasibility, acceptability, sensitivity, specificity, positive predictive value (PPV), negative predictive value (NPV) were investigated; the validity of the convergent construct was tested with self‐reported adherence measures and international normalized ratio (INR) stability.

## 2 METHODS

### 2.1 Design and setting

This was a methodological study outlined according to the COnsensus‐based Standards for the selection of health Measurement Instruments—COSMIN^25^ recommendations, and conducted in an OAC outpatient clinic of a large university hospital, in the interior of the São Paulo state, Brazil.

### 2.2 Sample

The study included 127 adult patients taking OAC in an outpatient follow‐up service. Patients who had been using OAC for at least 6 months were included. Patients whose OAC dosage was modified in the last month prior to the interview, who presented hemorrhagic or thromboembolic complications in the last 3 months, or who underwent surgery in the last 6 months prior to the interview were excluded.

### 2.3 Sample size

The sample size was calculated with the aim of estimating the sensitivity of the tool for assessing overall medication adherence. Sensitivity, in the present study, can be defined as the probability of an individual being classified as adherent by means of the global adherence assessment instrument, given that he/she has been classified as adherent by the gold standard instrument (The Morisky Medication Adherence Scale‐8 item). To carry out the sample calculation, estimates of sensitivity, specificity and prevalence were obtained from a pilot sample composed of 50 subjects. In addition, a significance level of 5%, a test power of 80% and a sensitivity value equal to 0.50 were established as a null hypothesis. From the pilot sample, estimates of sensitivity, specificity and prevalence equal to 0.65, 0.57 and 0.86 were obtained, respectively. The results indicated a sample of 105 subjects. Considering a loss rate of 20%, the final sample will consist of 127 participants.^26^, ^27^

### 2.4 Data collection procedure

Data were obtained by means of interview, using instruments. The results of the last three INR dosages, and the individual therapeutic goal recommended for each patient, were obtained from the medical record.

### 2.5 Instruments

Instrument of sociodemographic and clinical characteristics: was developed in a previous study^28^ and was submitted to content validity.

#### 2.5.1 Global Evaluation of Medication Adherence Instrument

**FIGURE 1:**
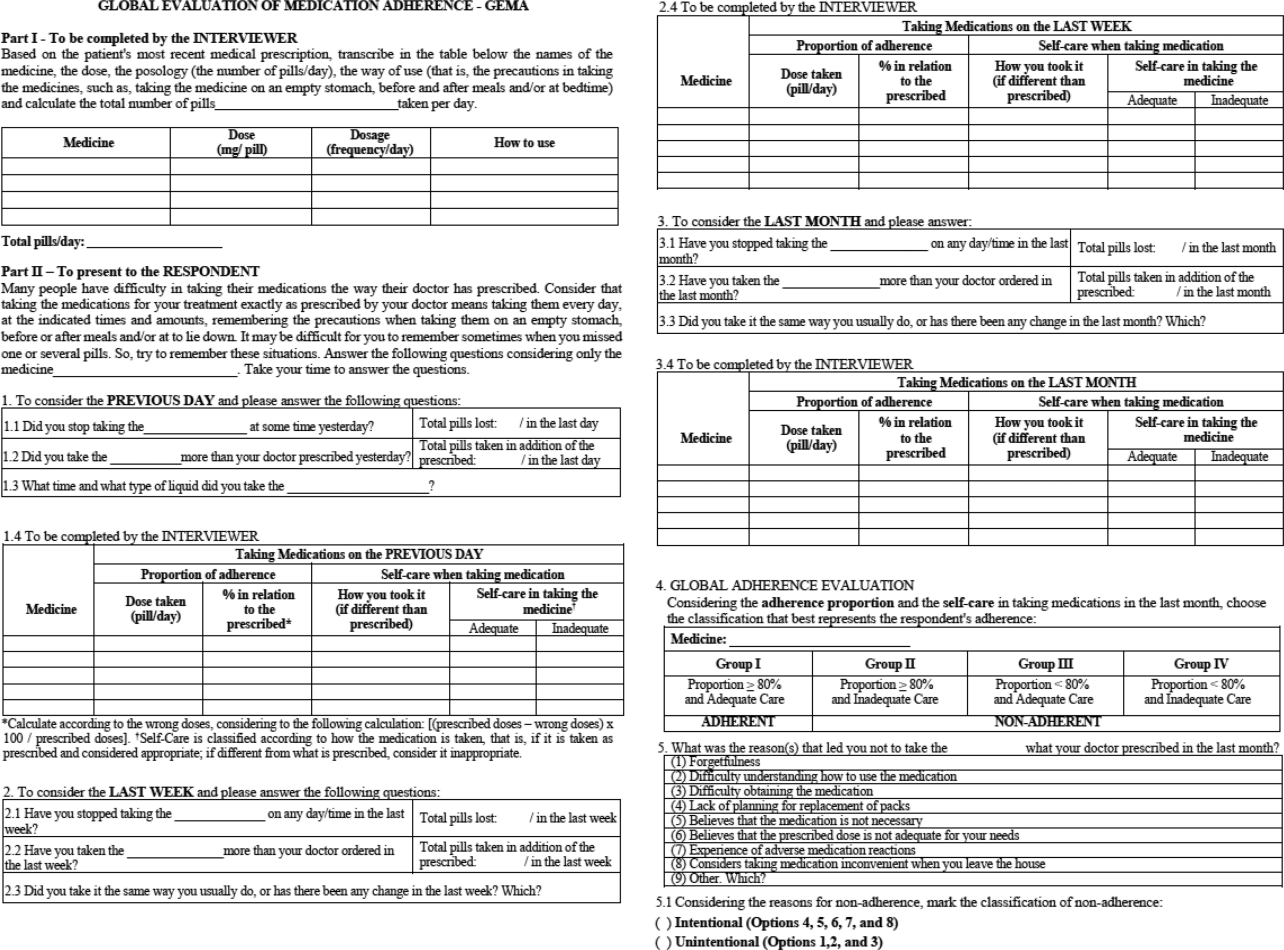
Global Evaluation of Medication Adherence Instrument (GEMA).

*Part I*: Completed by the interviewer, in order to transcribe the OAC prescribed, and in use by the patient, considering the dose, the dosing schedule (number of tablets/day), how to use it, (for the example, taking the medicine while fasting, before and after meals, and/or at bedtime), as well as calculating the total pills taken per day (Figure 1).

*Part II*: Composed of the objectives of the measure and by items which refer to the identification of erroneous doses (missed or over prescribed), care taken when taking the OAC, the proportion of adherence, and classification of care, in the past day, past week and past month. Items regarding medication intake in the previous day and week, were aimed at minimizing memory bias. The percentage of adherence to the prescribed dose is estimated considering the dose prescribed and the dose missed or taken beyond prescribed according to the calculation: ([prescribed doses−wrong doses] × 100/prescribed doses). Considering that the use of OAC than those prescribed does not result in adherence, the adherence results higher than 100% are converted by subtracting the percentage related to the over dosage, as shown below: 120% adherence, subtraction of the overdose (20%) resulting in adherence (100%–20% = 80%).^16^ The proportion of adherence is evaluated as a continuous categorical variable (adequate dose: ≥80% of agreement with the prescribed dose; inadequate dose: <80% of agreement with the prescribed dose). Self‐care includes the assessment of adequate dose implementation, schedule, time frames associated with taking the OAC, and adoption of care, according to the medication in use. The calculation of the Global Adherence Evaluation considers the Adherence Ratio and Medication self‐care in the last month for classification of patients into groups: I—Dose (≥80% of prescribed) and appropriate care prescription; II—Adequate dose and inadequate care; III—Inadequate dose (<80% of prescribed) and adequate care; and IV—Inadequate dose and inadequate care. Patients classified as Group I were considered to be adherent (Figure 1).

#### 2.5.2 The Morisky Medication Adherence Scale‐8 item

Is composed of eight items: seven with dichotomous answers (yes/no), and one Likert‐type item (“never”, “almost never” sometimes”,” often“ and “always”).^18^ The MMAS‐8 uses questions with inverted answers to reduce the social desirability bias, with the “no “ answer indicating the best adherence to six items, and the “yes” answer to a single item. The total score is calculated by summing the correct answers, ranging from 0 to 8, considering: high adherence (total score = 8), mean adherence (total score ≥6 and <8) and low adherence (total score <6). The MMAS‐8 was adapted and validated to the Portuguese language of Brazil,^29^ and in the Brazilian version, a total score = 8 was considered adherent, and a score <8 was non‐adherent. Its use was authorized by Donald E. Morisky, ScD, ScM, MSPH—Community Health Sciences, University of California, Los Angeles, USA. The Brazilian version of the MMAS‐8 was adjusted for OAC use, according previous study.^30^

#### 2.5.3 Measurement of Adherence to Treatments

The Measurement of Adherence to Treatments (MAT) consists of seven items that evaluate the daily behavior of medication intake, and whether the patient stopped taking the medications, for any reason. These items were adapted from other adherence measures. The Brazilian version of the MAT was adapted in patients using OAC,^30^ with a Likert‐type response scale (1 = “always”, 2 “almost always”, 3 = “frequently”, 4 = “sometimes”, 5 = “rarely” and 6 = “never”). The total score is obtained by the mean of the answers; the higher the score, the greater the adherence. The means with values between five and six were transformed into the value 01 (adherent) and means with values <5 were transformed into zero (non‐adherent). The Brazilian version of MAT showed evidence of reliability.^31^

#### 2.5.4 Stability of the INR

In order to determinate of the stability of the INR, three INR dosage results, performed up to 4 months prior to and on the day of the interview, were used to calculate INR stability^32^—the percentage of time in which the patients remained within the therapeutic range, according to the therapeutic goal for their clinical condition. Patients with at least 50% of the INR results within the therapeutic range were considered as having a stable INR. The remaining were considered as having an unstable INR.

### 2.6 Data analysis

Sociodemographic/clinical characteristics and adherence data were submitted to descriptive analysis. Friedman's ANOVA test was used to identify the differences in adherence ratios estimated by GEMA on the past month prior to the interview, using Dunn–Bonferroni post‐test to locate the differences. The McNemar's test was used to compare adherent and non‐adherent individuals the previous day, the past week, and the past month prior to the interview. A *p*‐value lower than .0167 was considering after applying the Bonferroni correction to the significance level in the McNemar's test. The Bonferroni correction in the significance level was applied because the same comparison was done in each of the three time points (previous day, the past week, and the past month). To the remaining tests the significance level was of 5%. The Shapiro–Wilk test was applied to evaluate the data distribution.^33^, ^34^

*Practicality and acceptability*: Evaluated by the mean time spent administering the questionnaire, and by the percentage of respondents who answered all the items, respectively.^33^

*Sensitivity, specificity, PPV and NPV*: The sensitivity and specificity of the GEMA, MMAS‐8 and MAT in relation to the INR stability, the clinical reference pattern for assessing the level of anticoagulation, were tested. Sensitivity was defined as the proportion of patients who were classified as non‐adherent in the instruments among all patients that were classified as having an unstable INR (by means of the INR stability criteria). Specificity was defined as the proportion of patients who were classified as adherent among all patients that were classified as having a stable INR. The PPV was defined as the proportion of patients who were classified as having an unstable INR among all patients that were classified as non‐adherent. The NPV was defined as the proportion of patients who were classified as having a stable INR among all patients that were classified as adherent.^27^, ^33^

*Construct validity*: It was tested considering the hypothesis that the global adherence measure provided by the GEMA and the measurements of the MAT and MMAS‐8 evaluate related but not identical constructs. This validity was estimated by the relationship between the percentage of doses (past month) of GEMA and MAT and MMAS‐8 scores. The Spearman correlation coefficient was used; the magnitude of the correlations was considered: weak for correlations close to 0.29; moderate for correlations between 0.30 and 0.49; and strong for those with correlations >0.50.^33^

Convergence construct validity was also tested by agreement between the GEMA—global evaluation adherence (percentage of doses and care taken in medication intake) and the adherence score obtained by the MAT and the MMAS‐8. It was assumed that the GEMA evaluates adherence based on the proportion of medication effectively taken according to medical prescription as well as on medication‐taking self‐care to classify patients into adherents and non‐adherents. MAT and MMAS‐8, in turn, are based on factors related to nonadherence to proceed the classification. Thus, as the tools are not measuring the same factors underlying adherence, agreement of weak or moderate magnitude were hypothesized between the classifications of adherents and non‐adherents by GEMA (past month), and those based on the MAT and the MMAS‐8. The Kappa coefficient was used, considering: poor agreement <.00; negligible = 0.00–0.20; weak = 0.21–0.40; moderate = 0.41–0.60; strong = 0.61–0.80; and almost perfect = 081–1.0.^34^

A significance level of 5% was adopted for these analyses.

## 3 RESULTS

The sample consisted of 50.4% women, married (63.8%), aged 56.5 (12.1) years, with 5.5 (3.7) years of study, unemployed (65.4%), and with a mean monthly family income of 2.6 (2.2). The sample presented about 1.9 (1.3) clinical conditions associated and mean time of anticoagulation was 55.5 (51.8) months.

### 3.1 Adherence and INR stability measure

The descriptive data for adherence measurements are presented in Table 1.

**TABLE 1:** Descriptive and comparative analysis of the Global Evaluation of Medication Adherence Instrument (GEMA), and the Brazilian version of Measurement Adherence to Treatments (MAT) and the Morisky Medication Adherence Scale‐8 item (MMAS‐8), when administered to patients using oral anticoagulants (*n* = 127).

	*n* (%)	Median	Q1–Q3^b^
GEMA^a^			
*Proportion of adherence*			
Previous day			
Adequate doses (≥80%)	123 (96.8)	100	100–100
Inadequate doses (<80%)	4 (3.2)		
Past week			
Adequate doses (≥80%)	120 (94.5)	100	100–100
Inadequate doses (<80%)	7 (5.5)		
Past month			
Adequate doses (≥80%)	121 (95.2)	100	96.5–100
Inadequate doses (<80%)	6 (4.7)		
Global evaluation of adherence^a^			
Previous day			
Adherent (Group I)	107 (84.2)**		
Non‐adherent (Groups II, III and IV)	20(15.8)		
Past week			
Adherent (Group I)	92 (72.4) *		
Non‐adherent (Groups II, III and IV)	35 (27.6)		
Past month			
Adherent (Group I)	83(65.3)**		
Non‐adherent (Groups II, III and IV)	44(34.7)		
Brazilian version of MAT			
Adherent	120 (94.5)	5.7	5.5–5.8
Non‐adherent	7 (5.5)		
Brazilian version of MMAS‐8			
Adherent	58 (45.7)	7.5	7.0–8.0
Non‐adherent	69 (54.3)		

^a^ Global evaluation of adherence considers the proportion of adherence and the care when taking medications.

^b^ Q1—first quartile; Q3—third quartile.

* *p* = .0027—McNemar's test.

** *p* < .0001—McNemar's test.

The values of adherence provided by GEMA presented a progressive reduction as the period of reference of the measure passed from the day prior the interview to the last past month. These differences were significant to the proportion of patients classified as adherents 84.2% (107); 72.4% (92) 65.3% (83) (*p* = .0027 and <.0001, respectively, McNemar's test).

The Brazilian version of the MMAS‐8 presented a median score of 7.5, which indicates nonadherence by the Oliveira‐Filho et al.^29^ classification. On the other hand, only 45.7% (58) were adherent according to one's classification. The MAT presented a median score of 5.7, which indicates adherence; and according to this scale, the majority of patients (94.5%) were adherent (Table 1).

Regarding the INR stability referring to the three measurements, more than half (55.7%) were considered unstable in relation to the individual therapeutic goal. The mean stability of INR was 44.1% (34.7).

### 3.2 Analyses of practicality, acceptability, sensitivity and specificity

Regarding practicality, the application of GEMA by interview took a mean time of 3.6 (1.6) min. Regarding acceptability, the rate of responses for the items was 100%.

The sensitivity and the specificity of the GEMA in the past month were tested against the INR, considering its stability in the last three measurements, according to the indicated therapeutic goal.

It was observed that GEMA is specific (0.76) for detecting the proportion of people who were adherent among those with a stable INR, but the GEMA is less sensitive (0.43) for detecting who was non‐adherent among those with an unstable INR. In other words, among the patients with a stable INR, most are assessed as adherent; among those with the non‐stable INR, although there are more adherents than non‐adherent's patients, there is a good concentration of non‐adherent patients. The GEMA also presented a PPV of 0.70 and NPV of 0.52.

It was verified that the performance of the GEMA is more consistent with the stability and instability results of the INR, when compared to the other adherence measures used in the present study. The MAT does not distinguish between adherence and non‐adherence among patients who present stable and unstable INR, as it classifies the expressive majority as adherents. The MMAS‐8 performs more similarly to GEMA, but among patients with stable INR, most patients are considered non‐adherent. The limits of both tools (MAT and MMAS‐8) in detecting those who were non‐adherent seems to be related to an overestimating of adherence in the studied sample (Table 2).

**TABLE 2:** Sensitivity and specificity tests, positive predictive value (PPV) and negative predictive value (NPV) of the Global Evaluation of Adherence Instrument (GEMA) and the Brazilian versions of the Measurement of Adherence to Treatment (MAT) and the Morisky Medication Adherence Scale‐8 item (MMAS‐8), and the stability of the international normalized ratio (INR) in patients taking an oral anticoagulant (*n* = 124).

	Stability of INR		Sensibility	Specificity	PPV	NPV
	Non‐stable	Stable				
GEMA						
Non‐adherent (Groups II, III and IV)	30	13	0.43	0.76	0.70	0.52
Adherent (Group I)	39	42				
MAT						
Non‐adherent (score <5)	5	2	0.07	0.96	0.71	0.45
Adherent (score 5–6)	64	53				
MMAS‐8						
Non‐adherent (score <8)	38	30	0.55	0.45	0.56	0.45
Adherent (score = 8)	31	25				

### 3.3 Convergent construct validity

The convergent construct validity was tested by correlation between the adherence ratio, estimated by proportion of doses of the GEMA, and the adherence scores obtained by MAT and MMAS‐8. Positive low magnitude correlations were expected between the percentages of doses obtained (the previous day, past week, and past month) and the adherence scores of the MAT and MMAS‐8 (Table 3).

**TABLE 3:** Spearman correlation coefficient (*r*) between the dose proportions obtained by the Global Evaluation of Medication Adherence Instrument (GEMA) and the Brazilian version of the Measurement Adherence to Treatment (MAT), and the Morisky Medication Adherence Scale‐8 item (MMAS‐8) in patients taking an oral anticoagulant (*n* = 127).

GEMA	Brazilian version—MAT (*r*)	Brazilian version—MMAS‐8 (*r*)
Adherence proportion		
Previous day prior to interview	−0.08	0.15
Past week prior to interview	0.22*	0.26*
Past month prior to interview	0.30**	0.22*

* *p* <.05

** *p* < .001

Significant positive correlations of moderate and weak magnitudes were found between the proportion of adherence in the past month (*r* = .30) and in the past week (*r* = .22), estimated by the GEMA and MAT scores. Correlations of low magnitude were identified between the proportion of GEMA adherence in the past week, and the total score of MMAS‐8 (*r* = .26), and in the month prior to the interview (*r* = .22; Table 4).

**TABLE 4:** Concordance coefficients between adherence classifications ratings (adherent and non‐adherent) obtained by the Global Evaluation of Medication Adherence Instrument—GEMA, the Measurement Adherence to Treatment (MAT) and the Morisky Medication Adherence Scale‐8 item (MMAS‐8), in patients taking oral anticoagulation (*n* = 127).

	GEMA		Kappa coefficient (CI)
	Non‐adherent	Adherent	
MAT			
Non‐adherent (score <5)	5	2	0.11 (−0.01; 0.23)
Adherent (score 5–6)	39	81	
MMAS‐8			
Non‐adherent (score <8)	31	38	0.22 (0.06; 0.37)
Adherent (score = 8)	13	45	

Abbreviation: CI, confidence interval.

The convergent construct validity was also assessed, considering the proportion of agreement between the measures of adherence (Table 4).

No agreement was found between the classification of the GEMA and MAT (Kappa = 0.11, CI = −0.01 to 0.23). The agreement among GEMA and the adherence classification of the MMAS‐8 was higher, according Landis & Koch,^29^ but not enough to be considered satisfactory (Kappa = 0.22, CI = 0.66–0.37). A higher proportion of adherent patients was found to be present with the MAT and MMAS‐8, when compared to the GEMA, a fact that can explain the low proportion of agreement between the instruments.

## 4 DISCUSSION

The study aimed to evaluate the properties of measurement of the GEMA instrument, when administered to outpatients in use of OAC.

The application of GEMA by interview seems to be relatively fast, even with the need of thinking about the use of the medication in three different periods, what reinforces the acceptability of the tool. The short time of application of instrument is an important aspect, especially in patients with chronic diseases, whose treatment involves several simultaneous evaluations. Consequently, the use of measurement with a long time of application can imply in the commitment of the dynamism in the care of these patients.

The rate of responses to the items was 100%, although it could be facilitated by the mode of interview. With the sample population, the interview was the choice for the application of the tool due to the low level of schooling. It would be interesting in further studies to analyze the potential of self‐administration of the tool.

Regarding, the GEMA sensitivity, specificity, PPV and NPV analyses, in relation to INR stability, considered as the clinical reference measure, the data showed that the GEMA is an instrument capable of identifying those who are adherent among those who had a stable INR. There was a limited ability noted to identify individuals who were non‐adherent among those with unstable INRs, which makes it possible to classify a large proportion of individuals with unstable INRs as non‐adherent (PPV = 0.70).

However, the GEMA presents an overall better performance to the Brazilian versions of MAT and MMAS‐8, considering that MAT seems to overestimate adhesion, while MMAS‐8 overestimates non‐adhesion, and a possible explanation is that both instruments (MAT and MMAS‐8) do not consider self‐care, which includes the assessment of adequate dose implementation, schedule, time frames associated with taking the OAC, and adoption of specific care. On the other hand, the MMAS‐8 showed a slightly higher sensitivity to INR stability than the GEMA, that is, a better ability to identify those who were non‐adherent among individuals with unstable INR, but with limited capacity to identify who was adherent among those with stable INRs.

A Korean validation study of the MMAS‐8, which adherence to antihypertensive medication was classified as low (score <6) and medium/high adherence (score ≥6), showed sensitivity, specificity, PPV and NPV in relation to blood pressure measurement of 64.3%, 72.9%, 29.5% and 92%, respectively. However, when the classification of low and medium adherence (score <8) and high adherence (score = 8) were used, the MMAS‐8 presented sensitivity, specificity, PPV and NPV of 82.1%, 36.9%, 18, 7% and 92.1%, respectively.^35^ These results corroborate the findings in our study, in relationship to the higher sensitivity and lower specificity of the measure, against changes in the classification of the MMAS‐8 response, that is, by changing the cutoff point from six to eight, to classify the high adherence.

In contrast, in the MMAS‐8 validation study performed in Singapore that considered those with a score <8 to be non‐adherent, and which used the time in therapeutic range (TTR) of the INR as the gold standard, the sensitivity, specificity, PPV and NPV results were 73%, 35.6%, 49.5% and 60.5%, respectively,^30^ which differs from the results of our study, possibly due to the use of the 80% TTR as a desirable anticoagulation control.

GEMA performs better probably because it considers factors that describe behavior more than the other two measures, which are based on factors that influence adherence and the establishment of arbitrary cutoff points.

The convergent construct validity of the GEMA, tested by means of the ratio between the proportion of doses (obtained after memory retrieval at different time points—previous day, past week, and past month), and the MAT and MMAS‐8, showed significant positive correlations between low and moderate magnitude between the estimated proportion of GEMA in the past month prior to the interview, and the MAT and MMAS‐8 scores, partially supporting the validity convergent construct of the GEMA.

As to the agreement between the global adherence (proportion and care) of the GEMA and the Brazilian versions of the MAT and MMAS‐8, used to test validity, showed a negligible agreement with the MAT and weak agreement with the MMAS‐8, according to the Brazilian classification, which corroborates the hypothesis that the MAT and MMAS‐8 instruments measure related constructs, but not concepts that are identical to the GEMA adherence measure. The MMAS‐8 items evaluate different aspects related to nonadherence, while the GEMA, when measuring the percentage of doses and the care taken in the medication, deviates the focus away from nonadherence factors. In fact, these factors are investigated among patients classified as non‐adherent, but are not considered in the measurement of the adherence measure provided by the GEMA, which may have contributed to the poor agreement obtained among the instruments.

The findings obtained with the use of GEMA suggest that the search in one's memory for the behavior of adhering to medications at different moments in time, that is, from the day before the interview to the past month (interest evaluation), enable more accurate measurement, evidencing an important role of the GEMA in reducing memory bias in adherence measurement.

Gagné and Godin consider that memory bias should be minimized by specifying the period of time for questions that investigate nonadherence.^36^ Short periods would be related to episodic memory (specific episodes of the behavior), while long periods would be related to semantic memory (generalizations of behavior),^37^, ^38^ and it would be necessary to find an ideal period of time for investigation of the adherence behavior that is not too close or too far from the date of interest of the adherence assessment.^39^ Thus, it seems important to question from the most recent period to the furthest period, even if the most recent period is only to reduce memory bias, and does not represent adherence in the period of interest.^39^

The measurement provided by GEMA has potential applications for clinical and research practice. With regard to clinical implications, this instrument can be used to identify specific situations in different chronic conditions, related to the proportion of adherence and self‐care in taking medication as prescribed by the physician, thus enabling health professionals to direct actions toward promoting adherence to pharmacological treatment.

As a research tool, the medication adherence construct provided by GEMA could be a valuable variable of outcome, which could be measured over time in response to a behavioral, cognitive or educational intervention, providing evidence on the effectiveness of different strategies for promoting health. Thus, the instrument can be used in studies that aim to deepen knowledge about the mediating and/or moderating variables of this complex behavior.

In the present study, limitations such as the administration of the GEMA by means of interview can contribute to overestimation of adherence; the small sample size may also have influenced the findings. We recommend continuity of investigation on the GEMAS's measurement properties, especially the refinement of its validity, analyzing the relationship with direct measurement of adherence—concentration of the medicine or its metabolite in body fluids and/or the use of biological markers.

## 5 CONCLUSION

In conclusion, GEMA is a practical instrument, easy to administer by means of interviews, with little time and resources required for its administration. In addition, this measurement presents evidence of acceptable, sensitivity and specificity considering self‐reported measures of adherence available in the Brazilian culture (MMAS‐8 and MAT). The GEMA is a sensitive and specific tool regarding the stability of the INR. Construct validity was partially supported by significant positive correlations of low to moderate magnitude between the mean proportions of doses of the GEMA and scores of the MAT and MMAS‐8.

## AUTHOR CONTRIBUTIONS

Mariana Dolce Marques, Rafaela Batista dos Santos Pedrosa, Henrique Ceretta Oliveira, Maria Cecília Bueno Jayme Gallani, Roberta Cunha Matheus Rodrigues participated in research conception and design. Mariana Dolce Marques, Rafaela Batista dos Santos Pedrosa, Henrique Ceretta Oliveira collected and assembled the study data, and execute the study including informed consent. Mariana Dolce Marques, Rafaela Batista dos Santos Pedrosa, Henrique Ceretta Oliveira, Maria Cecília Bueno Jayme Gallani, Roberta Cunha Matheus Rodrigues considered the study result and method of analysis. Mariana Dolce Marques, Rafaela Batista dos Santos Pedrosa, Henrique Ceretta Oliveira, Maria Cecília Bueno Jayme Gallani, Roberta Cunha Matheus Rodrigues provided final approval of the manuscript. Henrique Ceretta Oliveira performed the statistical analysis. All authors read and approved the final manuscript.

## DISCLOSURE

The authors report no conflicts of interest in this work.

## ETHICS STATEMENT

The authors confirm that the research carried out in this study was conducted in accordance with the ethical guidelines and regulations of their institution. The study was approved by the Ethics Committee of the University in the state of São Paulo, Brazil (document no. 928.775) and the enrolled patients signed the Informed Consent Form. Confidentiality and anonymity of the participants were maintained throughout the study, and any personal information was protected in accordance with institutional policies and regulations.

## Data Availability

The data that support the findings of this study are available from the corresponding author, RBSP, upon reasonable request.
